# Immobilization of Trypsin in Lignocellulosic Waste Material to Produce Peptides with Bioactive Potential from Whey Protein

**DOI:** 10.3390/ma9050357

**Published:** 2016-05-12

**Authors:** Juliana Cristina Bassan, Thaís Milena de Souza Bezerra, Guilherme Peixoto, Clariana Zanutto Paulino da Cruz, Julián Paul Martínez Galán, Aline Buda dos Santos Vaz, Saulo Santesso Garrido, Marco Filice, Rubens Monti

**Affiliations:** 1 Faculdade de Ciências Farmacêuticas, UNESP—Univ. Estadual Paulista, 14800-903, Departamento de Alimentos e Nutrição, Araraquara-SP, Brazil; julianacbassan@gmail.com (J.C.B.); jpaulmg1@yahoo.es (J.P.M.G.); 2 Instituto de Química, UNESP—Univ. Estadual Paulista, 14800-060, Departamento de Bioquímica e Tecnologia Química, Araraquara-SP, Brazil; milenatsb@gmail.com (T.M.d.S.B.); clarianadacruz@gmail.com (C.Z.P.d.C.); alinebuda@zootecnista.com.br (A.B.d.S.V.); saulosantesso@iq.unesp.br (S.S.G.); 3 Faculdade de Ciências Farmacêuticas, UNESP—Univ. Estadual Paulista, 14800-903, Departamento de Bioprocessos e Biotecnologia, Araraquara-SP, Brazil; 4 Fundación Centro Nacional de Investigaciones Cardiovasculares Carlos III, Melchor Fernández Almagro, Madrid 28029, Spain; marco.filice@cnic.es

**Keywords:** corn cob powder functionalized, trypsin, immobilization, reactor, whey protein hydrolysates, peptides

## Abstract

In this study, trypsin (Enzyme Comission 3.4.21.4) was immobilized in a low cost, lignocellulosic support (corn cob powder—CCP) with the goal of obtaining peptides with bioactive potential from cheese whey. The pretreated support was activated with glyoxyl groups, glutaraldehyde and IDA-glyoxyl. The immobilization yields of the derivatives were higher than 83%, and the retention of catalytic activity was higher than 74%. The trypsin-glyoxyl-CCP derivative was thermally stable at 65 °C, a value that was 1090-fold higher than that obtained with the free enzyme. The trypsin-IDA-glyoxyl-CCP and trypsin-glutaraldehyde-CCP derivatives had thermal stabilities that were 883- and five-fold higher, respectively, then those obtained with the free enzyme. In the batch experiments, trypsin-IDA-glyoxyl-CCP retained 91% of its activity and had a degree of hydrolysis of 12.49%, while the values for trypsin-glyoxyl-CCP were 87% and 15.46%, respectively. The stabilized derivative trypsin-glyoxyl-CCP was also tested in an upflow packed-bed reactor. The hydrodynamic characterization of this reactor was a plug flow pattern, and the kinetics of this system provided a relative activity of 3.04 ± 0.01 U·g^−1^ and an average degree of hydrolysis of 23%, which were suitable for the production of potentially bioactive peptides.

## 1. Introduction

One of the most important factors in enzyme immobilization technology is the support material because its characteristics determine the performance of the complex enzyme-support. The study of alternative supports for enzyme immobilization poses a challenge for the process because the support material must have physical resistance, hydrophilicity, ease of derivatization, resistance to biological degradation, a low cost of acquisition and high interaction between the enzyme–support per unit of weight [1]. Agroindustrial byproduct has great potential as an alternative technology in enzyme immobilization because of its physical characteristics, chemical composition and low cost, which is one of the most attractive aspects for industrial processes [2,3,4]. Agriculture and the subsequent processing of agricultural products generates a million tons of lignocellulosic byproducts annually, such as rice straw, peanut and soy hulls and sugarcane bagasse that have already been employed as supports. After chemical or biotransformation processes, these byproducts are considered as large sources of renewable energy (2G ethanol), low cost and a potential for application as new materials for environmentally friendly processes in chemical and food industries [5].

Corn cobs are generated as a byproduct of corn processing. Corn production is one of the most important agricultural activities worldwide [6]. Conventionally, corn cobs are an agricultural material that are employed as animal feed supplements and are used in organic fertilizer [7,8]. Brazil is the third largest producer of corn, producing 52 million tons of corn in 2012 [9]. Generally, each kilogram of milled dry grain generates 0.15 kilograms of corn cobs [10,11,12]. The high cost of commercial immobilization supports has encouraged the search for low cost alternatives [4]. Among low cost alternatives, corn cobs have useful physical–chemical characteristics for the production of many value-added substances for food, energy and chemical and pharmaceutical industries [13,14]. The immobilization of enzymes in a lignocellulosic support has been reported in studies on rice straw for the immobilization of invertase [15], laccase immobilized in spent grain [16], lipase in coconut fiber [17], trypsin in granular waste from a brewery [18] and laccase in coconut fiber [19]. Enzyme immobilization has the potential to enhance the performance of many processes because it allows reuse cycles in enzymatic reactors and promotes easy recovery and purification of products. Moreover, immobilized enzymes have increased stability, and their catalytic effect is maintained for a longer time [20,21,22]. An efficient technique is immobilizing an enzyme in a solid support by multipoint covalent bonds, which prevents conformational changes in the tertiary structure of the enzyme [23].

Trypsin (EC 3.4.21.4) is a serino-protease endopeptidase that has a high capacity of hydrolysis for most proteins, so it is largely used as a chemical reagent in proteomics (specific diagnosis biomarkers and highly efficient proteolysis of complex proteomic samples) [24], digestive insufficiency therapy [25], the chemical industry (organic synthesis) [26,27] and in the production of bioactive peptides from milk proteins such as caseins, because they are an important source of biologically active peptides [28]. Studies have demonstrated that bioactive peptides obtained from cheese whey are capable of affecting physiological responses in animals. Among such responses, the most important are the insulinotropic effect, antioxidant activity, immunomodulatory effects, opioid activity and anticarcinogenic and antihypertensive activities [29,30,31,32,33].

In this context, the purpose of this study was the evaluation of low cost supports in the development of a continuous process for the potentially bioactive peptides production from cheese whey.

## 2. Results and Discussion

### 2.1. Pretreatment and Characterization of Corn Cob Powder

Two pretreatments, a physical–chemical (lignol process with fast decompression) and a chemical (alkali) treatment, were considered to decrease the lignocellulosic biomass recalcitrance via the delignification and partial solubilization of the hemicellulose to achieve higher porosity, specific surface area and expose reactive groups [34,35,36], which allowed high immobilization yield and stabilization factors, as demonstrated by [19].

The pretreatments induced a modification of the morpho-chemical structure of the CCP (corn cob powder) due to hydrolysis that promoted effective lignin removal and the saponification of the ester bonds between lignin and the hemicelluloses [35]. The chemical composition of the CCP (Table 1) after the pretreatment indicated a total lignin reduction of ~67% (g/100 g) of the initial CCP.

**Figure 1:**
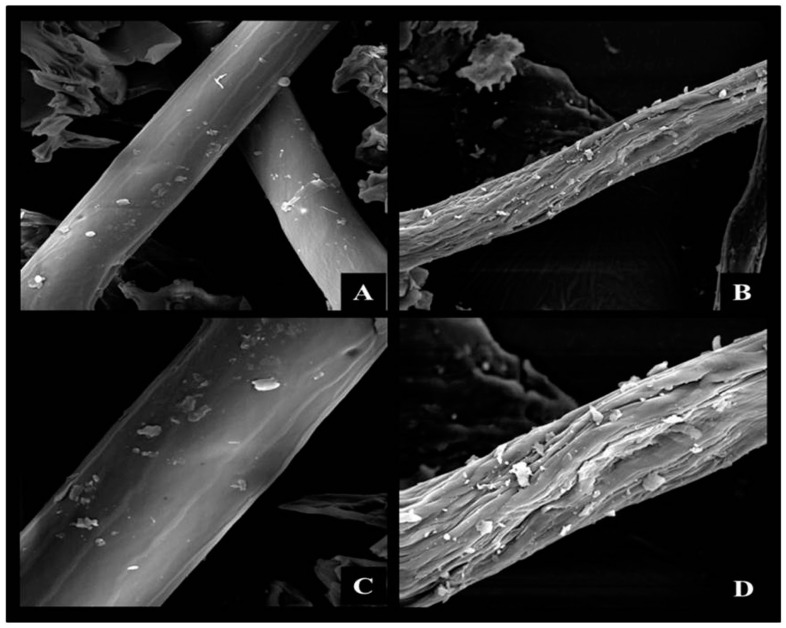
Scanning electron microscopy of corn cob powder after physicochemical pretreatment: Natural fiber 1000× (**A**) and 2000× (**C**); Thermal decompression in 70% ethanol + 2 M NaOH (8 °C/24 h in slow stirring) 1000× (**B**) and 2000× (**D**).

Partial hydrolysis of the hemicellulose also occurred, in terms of xylan, arabinoxyl groups and acetyl groups, with a dry weight reduction of 53.8%. Generally, an alkali acts effectively at the amorphous sites (lignin and hemicellulose), hydrolyzing ester bonds, acetyl groups, uronic groups, *etc.* [35,37]. The glucan fraction was less affected after the pretreatments. The extractives (sugars, waxes, nitrogenous compounds) [36] that accounted for 6.12% of the total CCP dry weight were also removed with high efficiency (Table 1 and Figure 1).

According to Figure 1, the combination of the pretreatments removed a major portion of the homogeneous outer layer that corresponded to the waxy fraction (Figure 1A–C) resulting in an irregular structure that had fissures all over its extension (Figure 1B,D), which improved access to reactive groups in the monomeric units that composed the polymeric structure of the cellulose and hemicellulose.

**Figure 2:**
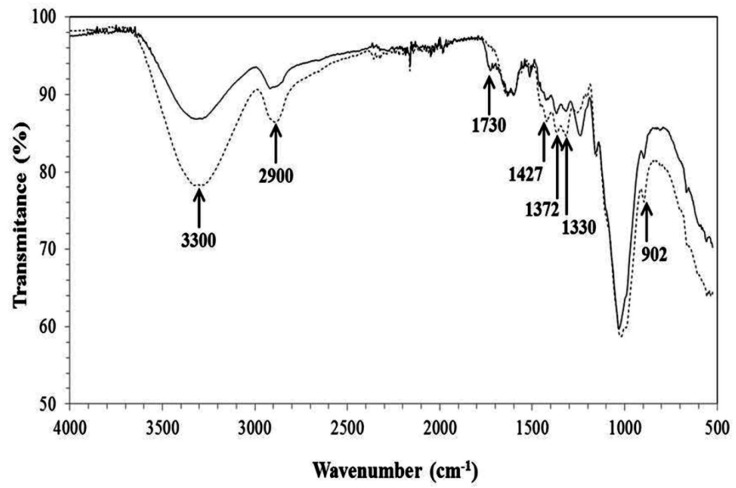
Infrared analysis of corn cob powder (CCP). Non-treated CCP (solid line) and pretreated CCP (dotted line).

The FTIR spectrum of the non-treated and pretreated CCP (Figure 2) suggested the occurrence of major modifications related to lignin and hemicellulose that corroborated the results obtained for the chemical composition (Table 1).

The decrease of peak at 902 cm^−1^ suggests a slight removal of amorphous cellulose [36,38] that may have reflected in the reduction of the C–O–C β-glycosidic bonds, and the peak at 1730 cm^−1^ represents the C=O groups of hemicellulose, which disappeared after the pretreatments, indicating the partial solubilization of this polymer [39]. A reduction in the absorption related to a peak length of 3400–3200 cm^−1^ also occurred, which corresponds to the stretching of O–H, indicating a rupture of the H bonds of cellulose [36] and the solubilization of the monomeric unities of hemicellulose. Another modification was observed in the peak at 2900 cm^−1^ (stretching of C–H), indicating the rupture of the methyl/methylene groups of cellulose [36,38]. The peak at 1427 cm^−1^ was also reduced after the pretreatments due to the vibrations of the aromatic rings combined with the planar deformation of the C–H of lignin [19,38]. The peaks at 1330 and 1372 cm^−1^ also indicated lignin removal because they represented the O–H phenolic and the flexing of C–H in –C–CH_3_, respectively [38].

**Figure 3:**
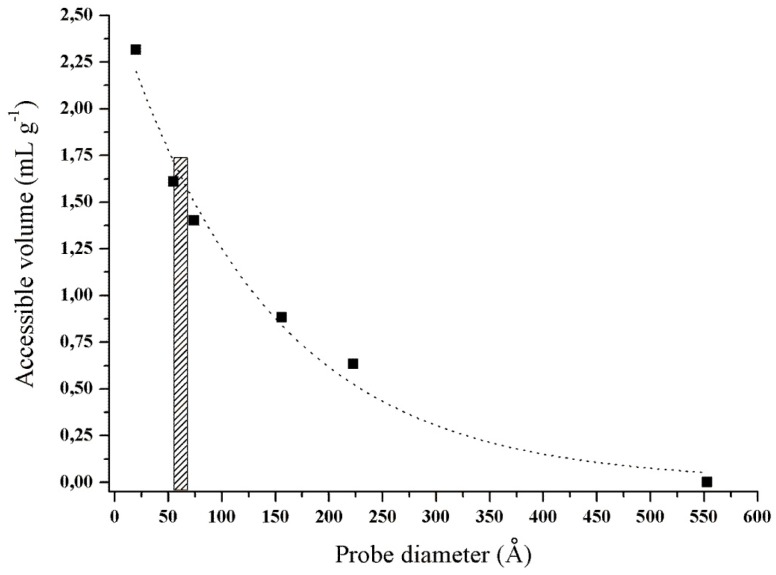
Porosity of the pretreated corn cob powder. The accessible volume per gram of CCP was performed by a solute exclusion technique. Six probes with sizes between 20 and 553 Å were used.

Regarding the porosity (Figure 3), the volume of pores accessible to the enzyme and the pore size distribution were determined by the diffusion of probes with different diameters (20 to 553 Å) inside the lignocellulosic matrix. The porosity of the pretreated CCP presented an accessible volume of 1.57 mL·g^−1^ to trypsin molecules, which corresponded to an orthorhombic structure with the dimensions of a = 54.84, b = 58.61 and c = 67.47 Å [40]. We inferred that pretreated CCP geometry of this pore distribution/dimension allowed the immobilization of trypsin inside of and on the surface of the pore.

### 2.2. Immobilization and Biochemical Characterization of Trypsin

The results presented in Table 2 shows that the immobilization rate for all of the activated supports was higher than 80%, and the CCP supports had the best performance. An exception occurred with IDA-glyoxyl-CCP, possibly due to the lower number of reactive groups in that support. After activation with glyoxyl groups, the titration of the aldehyde groups resulted in 97 µmols/g agarose and 150 µmols/g corn cob powder.

For the IDA-glyoxyl-AG and IDA-glyoxyl-CCP, we obtained 80 µmols/g and 195 µmols/g, respectively. Of the total amount of epoxy groups generated for both supports, approximately 90% were oxidized to glyoxyl groups. The activation of the CCP support is likely related to the hydrophilic characteristic of vegetal fibers due to the high density of hydroxyl groups that are distributed along the surface of these materials [41], which are necessary for the chemical activation and immobilization of enzymes. The removal of lignin and the partial removal of the hemicellulose (Table 2) resulted in the breakdown of the polymeric matrix (Figure 1), providing higher accessibility to the targeted chemical groups. In this study, we obtained activated CCP-supports that were highly efficient for the immobilization of trypsin, and similar activities to AG of the derivatives were recovered with levels above 73% (Table 2). The same range of activity was also found in the study by Marques *et al.*, 2011 [42]. The different derivatives and the free enzyme had a maximum activity at pH 9.0, except the trypsin-glutaraldehyde-CCP (pH 8.5) derivative. However, the temperature for maximum activity and the half-life of the derivatives showed differences regarding the type of support activation and the soluble enzyme (Table 2). One of the main objectives of this immobilization procedure was to achieve enzyme stability [43,44,45]. The multipoint covalent immobilization on the supports glyoxyl and IDA-glyoxyl caused the structural rigidification of trypsin [42,45], providing higher stability compared to the free enzyme [42]. In the specific case of trypsin, the lysine residues in the three-dimensional surface are deprotonated amino groups (pH 10.0) that covalently bind to aldehyde groups of glyoxyl and ida-glyoxyl carriers.

### 2.3. Thermal Stability of Derivatives

**Figure 4:**
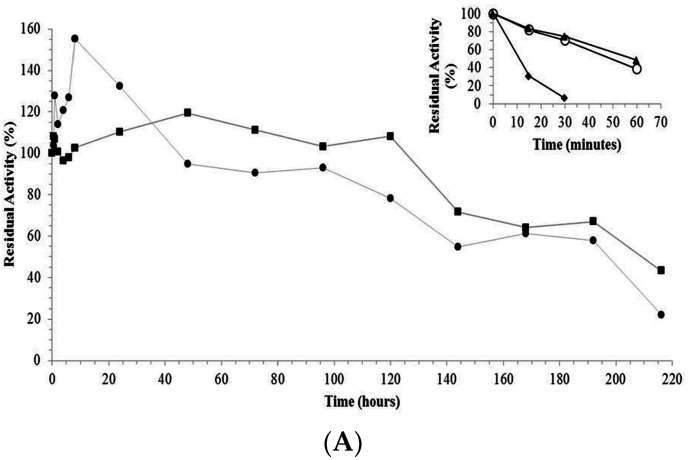

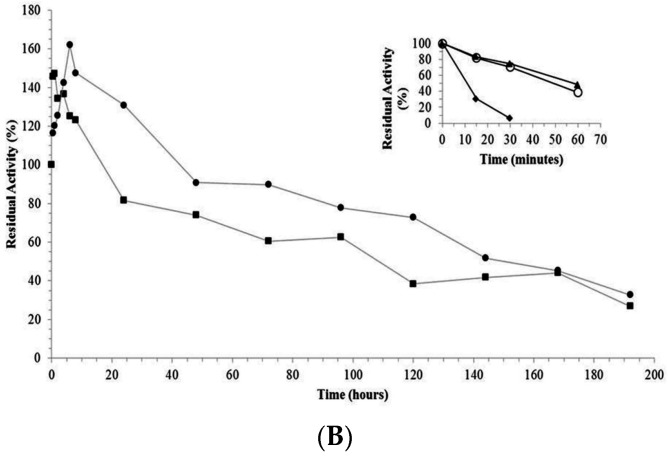
(**A**) Thermal stability of free trypsin and derivatives. (■) trypsin-glyoxyl-agarose derivative; (●) trypsin-glyoxyl-CCP derivative. The inner graph represents the time-course of thermal stability of (♦) free trypsin; (▲) trypsin-glutaraldehyde-agarose derivative; and (○) trypsin-glutaraldehyde-CCP-derivative. Experiments were carried out, independently, at 65 °C and pH 8.0. The initial activity was considered as 100% (t = 0). Measurements were performed at 25 °C and pH 9.0; (**B**) Thermal stability of free trypsin and derivatives. (■) Trypsin-IDA-glyoxyl-agarose derivative; (●) trypsin-IDA-glyoxyl-CCP derivative. The inner graph represents the time-course of thermal stability of (♦) free trypsin; (▲) trypsin-glutaraldehyde-agarose derivative; and (○) trypsin-glutaraldehyde-CCP-derivative. Experiments were carried out, independently, at 65 °C and pH 8.0. The initial activity was considered as 100% (t = 0). Measurements were performed at 25 °C and pH 9.0.

The trypsin-AG and trypsin-CCP derivatives had higher stability than the free enzyme after incubation at 65 °C and pH 8.0. The trypsin-glyoxyl-AG (1156-fold) and trypsin-glyoxyl-CCP (1090-fold) derivatives were the most stable, followed by trypsin-IDA-glyoxyl-CCP (883-fold), trypsin-IDA-glyoxyl-AG (600-fold), trypsin-glutaraldehyde-CCP (5.27-fold) and trypsin-glutaraldehyde-AG (4.36-fold), as demonstrated in Table 2 and Figure 4A,B.

**Figure 5:**
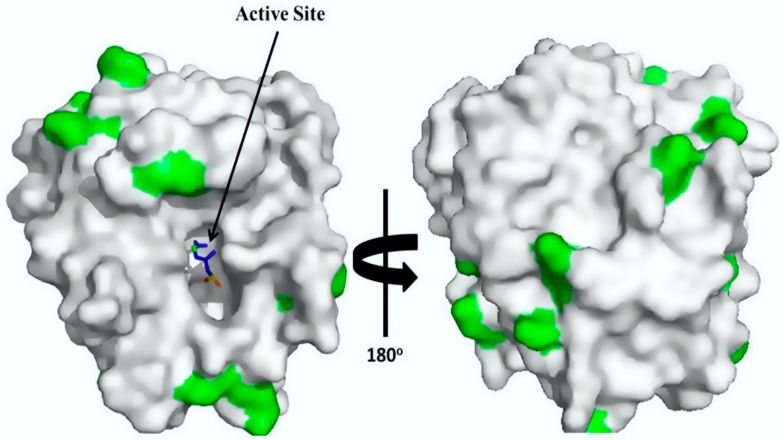
3D surface structure of trypsin (Protein Data Bank: pdb code 5ptp): trypsin structure with external lysine residues marked in green. The 3D surface structure of trypsin was obtained using PyMol 1.7.4.4 (Schrödinger, Tokyo, Japan).

The high stability of the trypsin-glyoxyl-AG and trypsin-glyoxyl-CCP derivatives is likely related to the presence of high density lysine residues in trypsin (Figure 5), in which the primary reactive amino groups (pH 10.0) allow the establishment of stable multipoint covalent bonds with reactive aldehyde groups on the surface of the support material [23]. Similar results were observed for the trypsin-IDA-glyoxyl-CCP and trypsin-IDA-glyoxyl-AG derivatives that had higher stability than the free enzymes. On the other hand, both trypsin-glutaraldehyde derivatives had the lowest thermal stability. This result is explained considering that immobilization via glutaraldehyde chemistry occurs at pH 7.0 involving the reactive terminal amino of the enzyme and the aldehyde groups of the support. In the case of trypsin, a multimeric enzyme, the terminal amino groups are exposed on different planes of the enzyme surface preventing the concomitant bonding of all amino groups on the support surface.

### 2.4. Application of Derivatives to Produce Peptides

**Figure 6:**
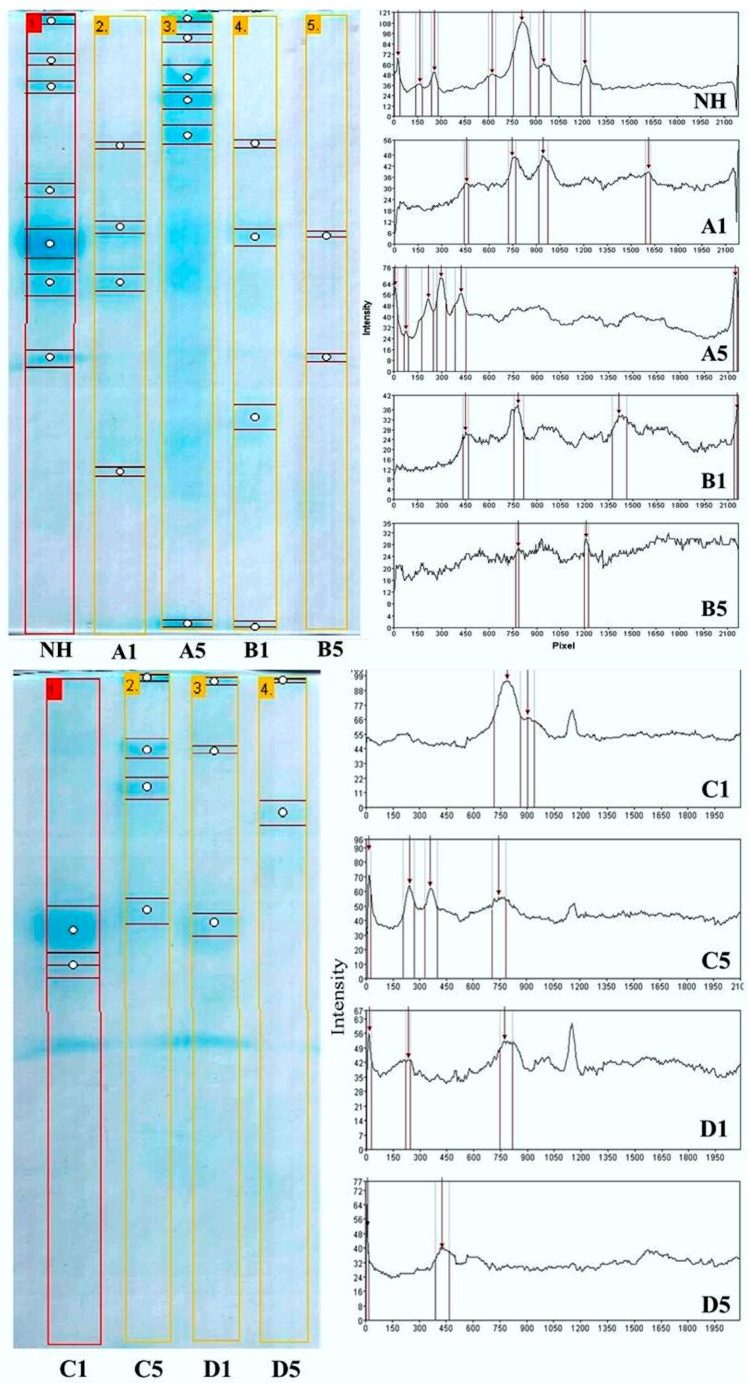
Hydrolysis profile of the cheese whey proteins performed by SDS-PAGE 12%. A comparison between the first and fifth cycles of reuse of stabilized derivatives: non-hydrolyzed whey protein (NH); trypsin-glyoxyl-AG (A1 and A5) and CCP (B1 and B5); and trypsin-IDA-glyoxyl-AG (C1 and C5) and CCP (D1 and D5).

**Figure 7:**
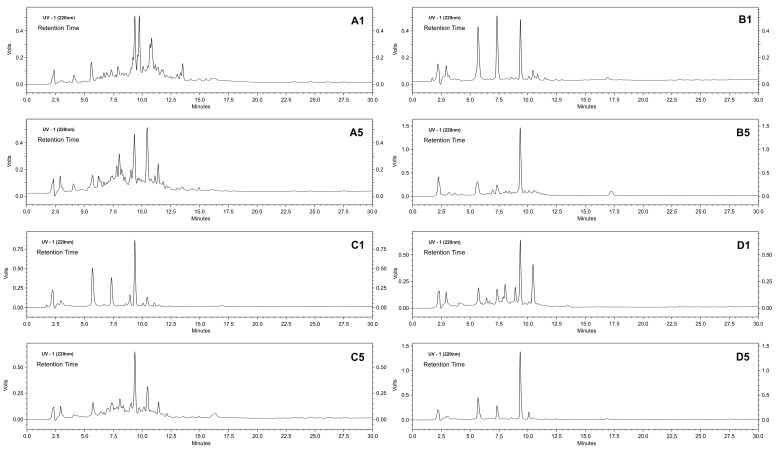
Hydrolysis profile of the cheese whey proteins performed by RP-HPLC. A comparison between the first and fifth cycles of reuse of stabilized derivatives of trypsin-glyoxyl-AG (A1 and A5) and CCP (B1 and B5); and trypsin-IDA-glyoxyl-AG (C1 and C5) and CCP (D1 and D5).

Figure 6 and Figure 7 show the electrophoretic and chromatographic profiles of the hydrolysates generated during the reuse of the derivatives (Cycle 1 through Cycle 5). The SDS-PAGE analysis confirmed the hydrolysis of cheese whey proteins in the batch process with different derivatives (trypsin-glyoxyl-AG/CCP and trypsin-IDA-glyoxyl-AG/CCP). Moreover, the gel analysis via the GelAnalyzer^®^ software revealed that all of the profiles (Figure 6: A1,A5, B1,B5, C1,C5 and D1,D5) were distinct, especially of the non-hydrolyzed cheese whey (Figure 6NH). These results indicated an effective catalytic activity intrinsic to all derivatives, including corn cob powder (CCP), which have been evaluated as alternative to the current immobilization supports.

**Figure 8:**
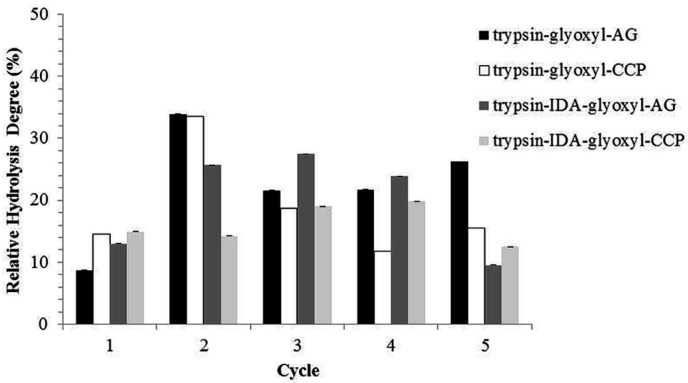
Reuse cycles with stabilized trypsin derivatives.

Figure 7 shows that different types of activation/immobilization produced protein hydrolysates with similar profiles, as demonstrated by the peaks observed with retention times of approximately 2.25, 5.75, 7.5, 9.5 and 10.5 min, which indicate that the enzyme specificity had not changed for the different derivatives. However, some specific characteristics in the hydrolysis profile were likely related to the support geometry and substrate diffusion. A larger difference was observed in chromatograms for Cycles 1 and 5 of the trypsin-glyoxyl-AG derivative (Figure 7A1,A5) that contained a large number of peaks between the retention times of two and 13 min. In addition, a complementary analysis of the hydrolysis degree also demonstrated these differences in the profiles in each reuse cycle (Figure 8).

These variations were probably related to mass transfer between the substrate and the immobilized enzyme, once substrate is composed of a mixture of proteins that have a molecular mass from 14.4 kDa to >97 kDa, thus preventing a homogeneous hydrolysis. The variation of mass transfer may have influenced the protocol utilized (TNBS), which depends on the reaction with α and ε-amino groups of the amino acids residues [46] released during hydrolysis. Despite this interference, the derivatives presented an effective hydrolysis degree at the 5 reuse cycles.

**Figure 9:**
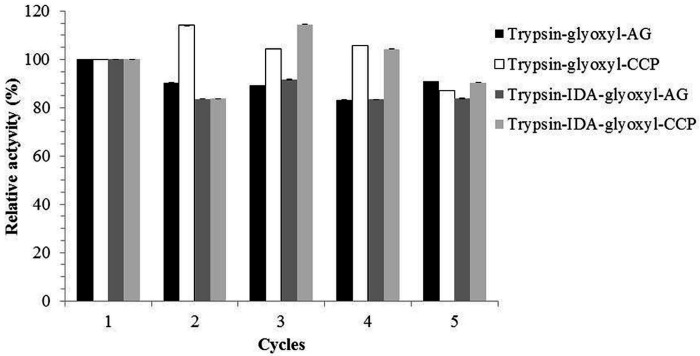
Relative activities of the stabilized trypsin derivatives after five reuse cycles.

The relative activity of the different derivatives was determined for each hydrolysis cycle (Figure 9). The variations in activity in each hydrolysis cycle were compared to the activity obtained in cycle 1, which was considered as 100%. In general, the derivatives showed a suitable retention of relative activity for five reuse cycles, with values above 80% compared to cycle 1.

### 2.5. Continuous Production of Peptides

**Figure 10:**
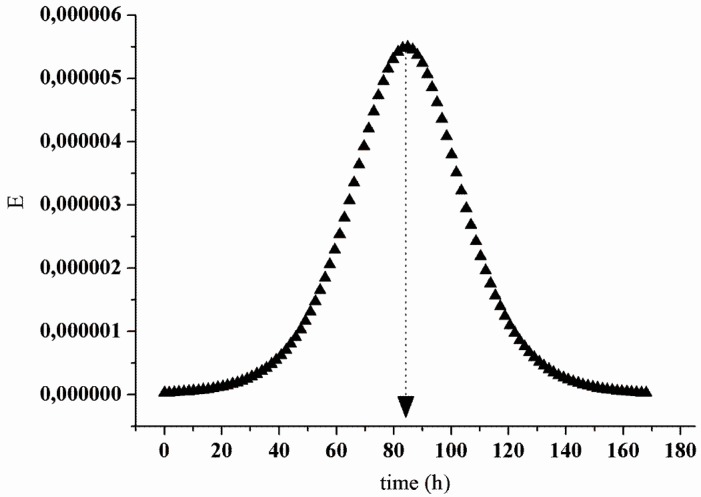
Response curve “E” for the hydrodynamic essay.

**Figure 11:**
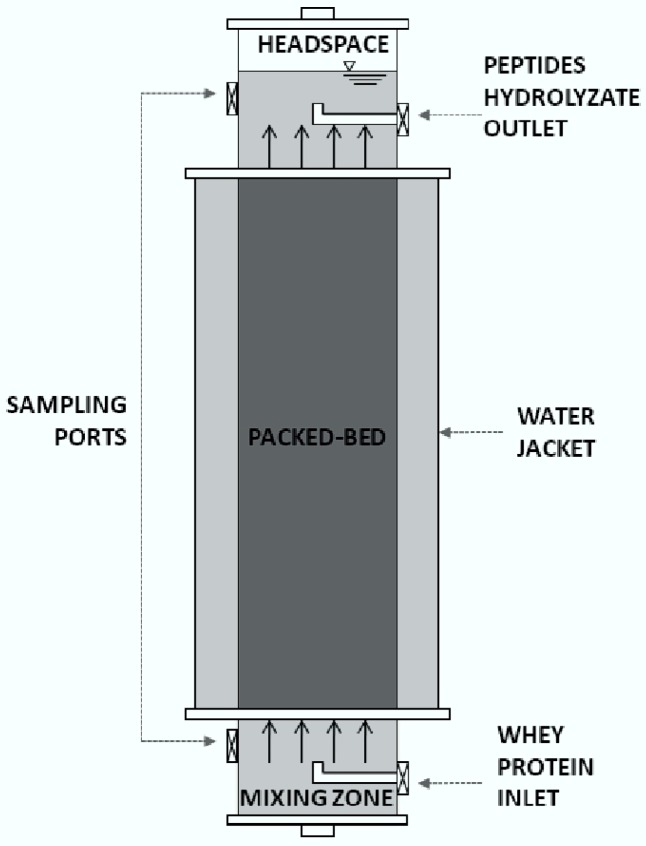
Upflow packed-bed reactor.

The characterization of the upflow packed-bed reactor (UPB) revealed that the actual hydraulic retention time (HRT) was 84.4 h. Figure 10 shows that this value was close to the theoretical HRT (78 h). It is worth mentioning that in the reaction compartment (packed-bed), the cheese whey proteins interacted with the derivatives for 42 h, which was the HRT for this reactor section (Figure 11).

For the experimental condition evaluated, the dispersion was 0.034 and the “n” of n-CSTRs model was 14.7, similar to the hydrodynamics of a plug-flow reactor (PFR). This hydrodynamic pattern (PFR) of the process is interesting because it corresponds to a series of continuous stirred tank reactors (CSTR) operating sequentially and results in a higher volumetric unit conversion than a single CSTR [47]. According to the profile shown in Figure 10 and the parameters (θh; D/μL; n) obtained in the hydrodynamic essay, there was no clogging of the packed-bed, flow channeling or a pressure drop in the system. Hence, the physical limitations of the UPB reactor did not have a negative effect on the process. Instead, the verification of the hydrolysis degree (HD) and relative activity (RA) indicated that the performance in continuous operation was further increased compared to the batch process.

The average hydrolysis degree in the batch process (five reuse cycles—Figure 8) was 18.8%, while in continuous operation (*i.e.*, in the UPB reactor) it was 23%. This difference is possibly related to an improvement of reaction conditions in the system due to the continuous removal of the reaction solution and avoidance of product inhibition. In terms of relative activity, the batch and continuous process presented 3.25 ± 0.03 U·g^−1^ and 3.04 ± 0.01 U·g^−1^, respectively. Such similar results were expected because the tests employed for the evaluation of the relative activity did not depend on the mass transfer characteristics. The continuous process was conducted for 99 h (four days) at a flowrate of 10.1 mL·h^−1^, resulting in a substrate loading rate (SLR) of 91.58 mg of protein per hour per liter of reactor. It is likely that operations with a higher SLR are perfectly feasible because a HD of 23% is considered high compared to others reported in the literature. In the studies by [48] utilizing soluble trypsin and [18] employing trypsin immobilized in spent grains, the HDs were 4.2% and 4.8%, respectively. Therefore, the use of immobilized enzyme in continuous processes is likely a critical step towards scale production of bioactive peptides.

## 3. Materials and Methods

### 3.1. Materials

Agarose 6BCL was acquired from GE Healthcare Bio-Sciences (Uppsala, Sweden) and corn cob powder from SAGRAN (Industry of Animal Feed Ingredients Ltd., Salto Grande, Brazil), Trypsin (EC 3.4.21.4), *N*-α-benzoyl-DL-arginine-p-nitroanilide (BAPNA), benzamidine hydrochloride, 2,3-epoxy-1-propanol, 1,2-ethanodiamine (EDA), glutaraldehyde, sodium periodate (NaIO_4_)*,* sodium borohydride (NaBH_4_), iminodiacetic acid (IDA) and 2,4,6-trinitrobenzene sulfonic acid (TNBS) were purchased from Sigma-Aldrich (St. Louis, MO, USA).

### 3.2. Preparation and Physicochemical Characterization of Pretreated Corn Cob Powder

***CCP pretreatment:*** the goal was to reduce the recalcitrance of lignocellulosic matrix exposing the monomer units (e.g., glucose). The hydroxyl groups present in these units were chemically modified to aldehyde groups allowing the multipoint covalent bonds between support and lysine sites of enzymes. Firstly, the lignocellulosic byproduct (CPP) suspended in organic solvent must be subjected to high temperature to promote intramolecular hydrolysis of lignin, intermolecular disruption between lignin and hemicellulose, as well as hydrolysis of glycoside bonds of hemicelluloses. After, the alkali hydrolysis must be performed to remove lignin by saponification of intermolecular ester bonds (hemicelluloses-lignin), which results in the increase of internal area and separation of structural connections. For this purpose, dried CCP was suspended in a 70% ethanol solution (1:10; *w*/*v*) and heated at 121 °C (1.3 Kgf/cm^2^) for 20 min, then rapidly decompressed [49]. CCP was repeatedly washed with deionized water and vacuum filtered. Next, the solid fraction was treated with alkali (2 mol NaOH) in a stirred suspension (140 rpm) for 24 h. Finally, the washing and vacuum filtering procedure was repeated.

***Chemical composition:*** The assessment was based on the quantification of lignin and sugars. Ethanol-soluble extractives were determined by extraction with 95% (*v*/*v*) ethanol in a Soxhlet apparatus. The samples were air-dried and milled to pass through a 0.84 mm screen. Approximately 3 g of the milled sample was extracted with 95% ethanol for 6 h in the Soxhlet apparatus. The percentage of extractives was determined on the basis of the dry weight of the extracted and non-extracted sample.

Ethanol-extracted lignocellulosic material samples were hydrolyzed with 72% sulphuric acid at 30 °C for 1 h at a ratio of 100:1 sample (mg) to sulphuric acid (mL). The acid was diluted with 79 mL of deionized water and the mixture was heated at 125 °C (1 atm) for 1 h. The residual material was cooled and filtered through a Number 3 porous glass filter. The solids were dried to constant weight at 105 °C and designated as insoluble lignin. The soluble lignin concentration in the filtrate was determined by the measurement of the absorbance at 205 nm. The concentration of monomeric sugars in the soluble fraction was determined by high-performance liquid chromatography (HPX87H column; Bio-Rad, Hercules, CA, USA) at 45 °C and an elution rate of 0.6 mL/min with 5 mmol/L sulphuric acid. Sugars were detected in a temperature-controlled refractive index detector at 35 °C (2414 Shimadzu-RID). Under these conditions, xylose, mannose and galactose eluted at the same time and appeared as a single peak. Glucose, xylose, arabinose and acetic acid were used as external calibration standards. No corrections were performed due to the sugar-degradation reactions that took place during acid hydrolysis. The factors used to convert sugar monomers to anhydromonomers were 0.90 for glucose and 0.88 for xylose and arabinose. The acetyl content was calculated as the acetic acid content multiplied by 0.72. This procedure was conducted in duplicate (the data were expressed as the mean ± SD) [50]. Glucose was reported as glucan after correction by the hydrolysis factor. The other sugars and acetic acid were used to calculate the non-cellulosic polysaccharide content [51].

***FTIR:*** the identification of the functional groups on the CCP surface was performed by Fourier Transform Infrared Spectroscopy with attenuated total reflectance. The readings were made in a Thermo Science Nicolet device. The analysis was conducted at CEMPEQ (Centro de Monitoramento e Pesquisa da Qualidade de Combustíveis, Biocombustíveis, Petróleo e Derivados, Araraquara, Brazil) of Chemistry Institute of Araraquara (IQ-UNESP).

***SEM:*** The analysis was performed on a TOP COM-SM-300 device at a voltage of 10–20 kV. The samples were prepared in a BAL-TEC SCD050 sputter coater. The gold sputter coating lasted 80 s at 25 °C and 41 mA in vacuum [19].

***Porosity:*** The solute exclusion technique was utilized, resulting in the ratio of pore volume per g of material. In our study, 0.1 g of dried CCP samples (105 °C) were added to six dextran probes (1.5%, m/m) of different molecular lengths (20–553 Å). The samples were kept in contact with the probes for 24 h at 25 °C. Subsequently, the samples were centrifuged for 15 min (12,000 *g*) and the supernatant was utilized to determine the final concentration of the probes in a High Performance Liquid Chromatograph (HPLC) equipped with Refractive Index Detector (RID). Deionized water was used as the mobile phase at 0.4 mL·min^−1^ and injection volume was 20 µL [51,52].

### 3.3. Activation of Corn Cob and Agarose Supports

***Glyceryl support:*** Each gram of CCP/AG was resuspended in 1.45 mL of deionized water, followed by the controlled addition of 2.38 mL 1.7 mol/L NaOH containing 0.068 g of sodium borohydride. The mixture was kept in an ice bath and 1.7 mL of glycidol was dropped in while the solution was agitated. The suspension was kept at 25 °C for 18 h and filtered. After filtration, the support was exhaustively washed with deionized water to remove soluble residual chemicals and neutralize the pH of the insoluble fraction [53].

***Glyoxyl support:*** To synthesize the glyoxyl supports, the pretreated CCP and AG supports were incubated with glycidol in order to generate the vicinal diol intermediate (glyceryl) on their surfaces. After, both supports were submitted to periodate-mediated oxidation, allowing the generation of aldehyde reactive groups on both surfaces and resulting in the glyoxyl-AG and glyoxyl-CCP. Each gram of CCP/AG-glyceryl was activated with glyoxyl groups via the addition of a solution containing 2 mL of 100 mmol/L NaIO_4_ and 8 mL of deionized water. The mixture was gently agitated for 2 h, and the support was separated and washed with deionized water [53]. The glyoxyl groups were quantified via iodometry [54].

***Amino support:*** To 1 g of CCP/AG-glyoxyl at pH 10.0, 5.71 mL of ethylenediamine (2 mol/L) was added, and the mixture was stirred (100 rpm) for 2 h at 25 °C. The remaining aldehydes were converted (reduced) to inert hydroxyls by adding 5.71 mg sodium borohydride to the mixture and stirring for 2 h. The amino support was filtered and washed with 10 volumes sodium acetate buffer (pH 4.0) and deionized water [55].

***Glutaraldehyde support:*** In total, 1 g of CCP/AG-amino was homogenized in a 2 mL 200 mmol/L phosphate buffer (pH 7.0) containing 3 mmol/L of glutaraldehyde. The suspension was gently agitated for 15 h at 25 °C. Subsequently, the support was filtered and washed with 20 volumes of 25 mmol/L phosphate buffer (pH 7.0) and deionized water [56].

***IDA-glyoxyl support:*** In the case of iminodiacetic aldehyde-activated carriers preparation (IDA-glyoxyl-AG and IDA-glyoxyl-CCP), both supports were firstly incubated with epychlorydrin in order to generate a reactive epoxy layer. After, the epoxy supports were submitted to partial acidic hydrolysis to generate, under controlled conditions, the vicinal diols necessary for the final generation of aldehyde groups. Subsequently, to link the IDA groups, both partially hydrolyzed supports were incubated overnight with an iminodiacetic acid solution in strongly basic conditions, allowing the reaction between the unreacted epoxy groups and iminodiacetic molecules. After washing, both supports were incubated with sodium periodate solution in order to generate the aldehyde groups, resulting in both IDA-glyoxyl heterofunctional supports used in trypsin immobilization. This heterofunctional support was prepared by a two-stage procedure. Stage 1: Activation with epoxy groups was done in a suspension of 1g of pretreated CCP and AG in 4.4 mL of deionized water, 1.6 mL of acetone, 1.1 mL of epichlorhydrin, 0.328 g of NaOH and 0.02 g of NaBH_4_. The suspension was gently shacked for 16 h, filtered, and washed with deionized water. The epoxy groups were then partially hydrolyzed incubating 1 g of support with 10 mL of a 0.5 mol/L H_2_SO_4_ solution for 1 h to hydrolyze the epoxy groups. To calculate the hydrolysis yield, a little amount of the hydrolyzed support (0.1 g) was oxidized with NaIO_4_ [53] and the amount of hydrolyzed epoxy groups was calculated as the difference between the periodate consumption by the hydrolyzed support and the same value retrieved using the initial epoxy support. The consumption of periodate was determined with iodometry [54]. Stage 2: Both epoxy supports were then treated with 0.5 mol/L iminodiacetic acid (pH 11.0) for 24 h at 25 °C [57]. Subsequently, filtration and washing were performed before final oxidation with 100 mmol/L NaIO_4_, according to the previously described protocol.

### 3.4. Preparation of the Trypsin Derivatives

***Trypsin-glyoxyl-AG/CCP:*** For each gram of activated support, 10 mL of 100 mmol/L carbonate/sodium bicarbonate buffer (pH 10.0) containing 5 mg of trypsin was added. The suspension was shaken for 24 h at 25 °C.

***Trypsin-glutaraldehyde-AG/CCP:*** For each gram of activated support, 10 mL of 50 mmol/L sodium phosphate buffer (pH 7.0) containing 5 mg of trypsin was added. The suspension was submitted to agitation for 2 h at 25 °C.

***Trypsin-IDA-glyoxyl-AG/CCP:*** This was a two-stage procedure. *Adsorption* (1): For each gram of activated support, 10 mL of 5 mmol/L sodium phosphate buffer (pH 7.0) containing 5 mg of trypsin (24 h agitation/25 °C) was added. *Covalent bounding* (2): The derivative proceeding from the previous step was suspended in 10 mL of 5 mmol/L carbonate/sodium bicarbonate buffer (pH 10.0) and agitated for 24 h (25 °C).

The immobilization of the different activated supports was performed in the presence of 3 mmol/L of benzamidine (a competitive inhibitor of trypsin) to prevent autolysis. In all cases, the immobilization efficiency was determined according to the measurement of remaining enzyme activity in the supernatant. After 80% of enzyme immobilization, the reaction was interrupted with the addition of sodium borohydride (1 mg/mL of solution) and gentle agitation for 30 min. The derivatives were washed with deionized water and stored properly prior to utilization.

***Determination of enzyme activity:*** The enzymatic activity was determined by a continuous colorimetric method by hydrolysis of BApNA at 405 nm (ε = 9.1 × 10^3^ M^−1^·cm^−1^ pH = 9.0) [58]. The free enzyme activity was measured in a 50 mM·L^−1^ carbonate/bicarbonate buffer system (pH 9.0) containing 0.2 mmol/L BApNA, 3.0 mmol/L benzamidine and 2% DMSO in a final volume of 2.2 mL at 25 °C. The activity of the derivatives was determined by the incubation of 10 mg in a 50 mmol/L carbonate/bicarbonate buffer system (pH 9.0) containing 0.2 mmol/L BApNA at 25 °C. One enzyme unit (U) was defined as the amount of enzyme necessary to produce 1 µM of p*-*nitroanilide per minute at the indicated temperature and pH 9.0 [42].

***Thermal stability:*** The soluble enzyme and the derivatives were incubated in TRIS-HCl 50 mmol/L buffer (pH 8.0) at 65 °C and the activity was monitored until 50% of the initial activity was decreased. The enzymatic activity was determined as described in Section 2.5.

### 3.5. Determination of Batch and Continuous Operation Parameters

#### 3.5.1. Reuse

A suspension of derivative (2.5 g) and 5 mL of cheese whey (pH 9.0) was incubated for 24 h in a water bath (45 °C) and gently agitated (140 rpm). At the end of each reuse cycle, the cheese whey was separated from the derivative by vacuum filtration and new substrate was added. Five reuse cycles were performed. The activity was monitored at the end of each cycle via the precipitation of whey proteins with trichloroacetic acid 10%. The absorbance of the supernatant (λ = 280 nm) was used in calculations, and 1 unit (U) of enzyme activity was defined as the amount of enzyme necessary to release 1 µM of tyrosine per minute [59].

#### 3.5.2. Whey Protein Hydrolysates Characterization

The stabilized derivatives (trypsin-glyoxyl-AG/CCP and trypsin-IDA-glyoxyl-AG/CCP) were used in the hydrolysis of cheese whey for up to 5 reuse cycles (45 °C/24 h per cycle). The profile of the products obtained with the different stabilized derivatives was qualitatively analyzed by SDS-PAGE, RP-HPLC, and the degree of hydrolysis was assessed with the trinitrobenzenesulfonic acid (TNBS) reaction and the relative activity via soluble protein precipitation with TCA.

***SDS-PAGE:*** Analyses on 12% SDS-PAGE [60] were performed to obtain the whey protein profiles and to assess protein hydrolysis. SDS-PAGE protein bands were stained with Brilliant Blue G-Colloidal (Sigma, St. Louis, MO, USA). The gel was analyzed with the GelAnalyzer^®^ software (LazarSoftware, Scottsdale, AZ, USA) for each reuse cycle performed in the batch process.

***Hydrolysis degree (HD):*** The HD was determined by the modified TNBS method from [61]. In this assay, 0.2 mL of sample or standard was added to 0.4 mL 5 mmol/L borate-NaOH buffer (pH 9.5) and 0.4 mL 5 mmol/L TNBS. The mixture was incubated at room temperature for 40 min. The reaction was stopped by the addition of 0.2 mL of 18 mmol/L Na_2_SO_3_ prepared in 2 mol/L NaH_2_PO_4_, and the absorbance was measured at 420 nm. *L*-Leucine at concentrations from 0.02–1.5 mmol/L was used as a standard, and the HD was determined by Equation (1):

(1) $$HD\left(\%\right)=100\frac{\left(AN_{2}-AN_{1}\right)}{Npb}$$

where *AN*_1_ is the amino nitrogen content of the protein substrate before hydrolysis (mg/g), and *AN*_2_ is the amino nitrogen content of the protein substrate after hydrolysis (mg/g). The nitrogen content of the peptide bonds in the whey (*Npb*) was 123.3 mg/g [62]. The values of *AN* were obtained by Equation (2):

(2) $$AN=\frac{ABS_{1}}{\left(\varepsilon .P\right)}$$

where *ABS* is the absorbance of the sample, ε is the molar extinction coefficient for *L*-Leucine (mg/L) and *P* is the protein concentration of the sample (g/L).

***RP-HPLC:*** The hydrolysates were filtered using a GV Millex 0.45 μm unit (Millipore, Darmstadt, Germany) and analyzed by RP-HPLC (Shimadzu LC 10A, Kyoto, Japan), which was equipped with a reverse phase Nucleosil C18 column (25 × 0.46 inch, 5 μm particle size, 300 Å pore size). The eluents of the mobile phase were as follows: (A) water and 0.045% trifluoracetic acid (TFA) and (B) acetonitrile (ACN) and 0.036% TFA with a 5%–95% linear gradient of eluent B, a flow rate of 1.0 mL/min for 30 min, with UV detection at 220 nm.

### 3.6. Characterization of the Continuous Process

An upflow packed-bed reactor (UPB) was employed to conduct the continuous process. This reactor had a length–diameter ratio (L/D) of 7.4 a height of 0.4 m and a working volume of 0.794 L. The packed bed occupied 58% of the total volume. The operation was conducted to provide a theoretical hydraulic retention time (HRT) of 78 h (39 h in the packed bed). For this purpose, the flowrate was adjusted to 10.1 mL/h. A schematic diagram of the system is shown in Figure 1.

Hydrodynamic assays were performed with a Labquest 2 interface coupled with a Vernier conductivity probe to detect the tracer concentration (10 g/L) in the reactor effluent. NaCl was used for a step stimulus assay [63] and monitored for 170 h (2 times the theoretical HRT). Data from the assays was processed with Microcal Origin 8.0^®^ to evaluate the real HRT (θh), the dispersion degree ($\sigma_{\theta }^{2}$) and the number of n-CSTR model [49] (N), utilizing Equations (3)–(5):

(3) $$\theta h=\frac{\int_{0}^{\infty }t.C\left(T\right).dt}{\int_{0}^{\infty }C\left(t\right).dt}$$

(4) $$\sigma_{\theta }^{2}=2\left[\frac{D}{µ.L}\right]$$

(5) $$\sigma_{\theta }^{2}=\frac{1}{N}$$

*C*, *F* and *E* curves were constructed using the experimental data [63]. In Equation (3), C is concentration and t is time. The variance of the curves ($\sigma^{2}$) indicated the dispersion of distribution and was calculated for the determination of dispersion number (D/µ.L) of the curves via Equation (4) (low intensity dispersions). The characterization of the hydrodynamic flow pattern was conducted according to the n-CSTR model presented in Equation (5).

The reactor performance was evaluated in terms of the substrate loading rate (Equation (6) and the HD (Equation (1)). Furthermore, the enzymatic activity [59] was also utilized to evaluate the feasibility of the continuous operation (Equation (7)).

(6) $$SLR=\frac{Q.S}{V}$$

(7) $$A=\frac{\sum_{abs}.1000.V_{t}}{\varepsilon \left(M\right).time.derivative}$$

In Equation (6), *Q* is the flowrate of the substrate (L/h), S is the substrate concentration (g) and V is the working volume of the reactor (L). In Equation (7), abs is the absorbance (nm), *V_t_* is the total volume of the assay and ε (*M*) is the molar extinction coefficient; the time is measured in minutes and the derivative is measured in grams.

## 4. Conclusions

A corn cob powder pretreatment for trypsin immobilization was successfully performed and allowed covalent binding inside and at the surface of the support pores. Moreover, the biochemical characterization of immobilized trypsin indicated that the trypsin-glyoxyl-CCP derivative had the highest performance for the immobilization yield (IY) and very similar results for the expressed activity (EA), half-lifetime (HF) and stabilization factor (SF) compared to those obtained with trypsin-glyoxyl-AG.

In the batch experiments, all derivatives (*i.e.*, trypsin-glyoxyl-AG/CCP and trypsin-IDA-glyoxyl-AG/CCP) effectively hydrolyzed the cheese whey with activities higher than 80% after 5 reuse cycles. Assays of the continuous experiments demonstrated a plug-flow hydrodynamic pattern, which was suitable for the process because of the high volumetric conversion of this hydrodynamic characteristic. The hydrolysis degree using trypsin-glyoxyl-CCP in this system was higher than that obtained in the batch experiments. In addition, the characteristics of reactor operation, like low dispersion (D/µ.L) and high number of n-CSTR model (N), indicated that the system was underutilized and higher flowrates and protein concentrations could be loaded.

Overall, the results of the batch and continuous experiments are collectively positive for future industrial application due to the development of the high performance immobilization of trypsin by low cost supports and their application in a suitable reactor configuration for the conversion of dairy waste into value-added products, such as bioactive peptides with antihypertensive, antimicrobial, antioxidative, antithrombotic and hypocholesterolemic properties.

## Figures and Tables

**Table 1:** Chemical characterization of pretreated corn cob powder.

Sample	Treatment Yield (%)	Chemical Composition ^a^						Mass Balance ^b^					
		Total Lignin	Glucan	Xilan	Arabinosyl Groups	Acetyl Groups	Extratives ^c^	Total Lignin	Glucan	Xilan	Arabinosyl Groups	Acetyl Groups	Extratives ^c^
Untreated	100	12.73 ± 0.03	28.02 ± 0.05	24.16 ± 0.16	7.15 ± 0.02	2.32 ± 0.02	6.12	12.73 ± 0.03	28.02 ± 0.05	24.16 ± 0.16	7.15 ± 0.02	2.32 ± 0.02	6.12
Steam/Alkali	52.6 ± 0.02	8.01 ± 0.18	47.62 ± 0.87	22.08 ± 0.20	7.43 ± 0.01	0.03 ± 0.03	0.97	4.21 ± 0.02	25.04 ± 0.03	11.61 ± 0.08	3.91 ± 0.01	0.01 ± 0.01	0.51

Note: ^a^ g/100 g of corn cob powder; ^b^ g/100 g of initial corn cob powder; ^c^ Ethanol soluble compounds.

**Table 2:** Immobilization parameters and physical properties of trypsin immobilized onto agarose and corn cob powder with different activations.

Derivatives	IY ^a^ (%)	EA ^b^ (%)	OpH ^c^	OT ^d^ (°C)	Half-Lifetime (h)	SF ^e^
Trypsin-glyoxyl-AG *	82.56	74.3	9.0	50	212	1156
Trypsin-glyoxyl-CCP **	85.34	75.4	9.0	60	200	1090
Trypsin-glutaraldehyde-AG	81.93	79.7	9.0	45	0.8	4.36
Trypsin-glutaraldehyde-CCP	94.61	74.2	8.5	45	0.97	5.27
Trypsin-IDA-glyoxyl-AG	84.70	73.1	9.0	55	110	600
Trypsin-IDA-glyoxyl-CCP	83.33	75	9.0	55	162	883

Note: * AG: agarose; ** CCP: corn cob powder; ^a^ Immobilization yield (IY): percentage of trypsin immobilized on different supports; ^b^ Expressed activity (EA): ratio between the activity in the derivatives obtained and the initial activity of the offered enzyme; ^c^ Optimum temperature; ^d^ Optimum pH; ^e^ Stabilization factor (SF): ratio between the half-life of the immobilized enzyme and that of the corresponding free enzyme.

## Abbreviations

The following abbreviations are used in this manuscript:

ACN	Acetonitrile
AG	Agarose
BApNA	*N*-α-benzoyl-DL-arginine-p-nitroanilide
CCP	Corn Cob Powder
CSTR	Continuous Stirred Tank Reactor
DMSO	Dimethyl Sulfoxide
EDA	1,2-ethanodiamine
FTIR	Fourier Transform Infrared Spectrometry
HRT	Hydraulic Retention Time
IDA	Iminodiacetic Acid
PFR	Plug Flow Reactor
SEM	Scanning Electronic Microscopy
SLR	Substrate Loading Rate
TFA	Trifluoracetic Acid
TNBS	2,4,6-trinitrobenzene Sulfonic Acid
